# Challenges in a six-phase process of questionnaire adaptation: findings from the French translation of the Integrated Palliative care Outcome Scale

**DOI:** 10.1186/s12904-019-0422-9

**Published:** 2019-04-18

**Authors:** Anca-Cristina Sterie, Mathieu Bernard

**Affiliations:** Palliative and supportive care service, Lausanne University Hospital and University of Lausanne, Avenue Pierre-Decker 5, 1011 Lausanne, Switzerland

**Keywords:** IPOS, Palliative care, Cross-cultural adaptation, Translation, Equivalence, French, Switzerland

## Abstract

**Background:**

The Integrated Palliative care Outcome Scale (IPOS) was developed for evaluating essential outcomes for palliative care patients. Our objectives here are to describe the process of a six-phase cross-cultural adaptation of IPOS to French (IPOS-Fr), highlight the difficulties encountered and strategies to solve them, and discuss the implications that adaptation may have on the validity and reliability of a questionnaire.

**Methods:**

The adaptation of IPOS consisted of six phases: (i) literature review and interviews with target population; (ii) forward translation to French; (iii) backward translation to English; (iv) Expert Review; (v) cognitive interviews with target population; (vi) final review.

**Results:**

Translation, cognitive interviews, and exchanges with Expert Review members allowed to make changes adapted to the target language regarding item 5 (“vomiting”) and 8 (“sore or dry mouth”), and to identify and address, in the original version of IPOS, syntactic inconsistencies in language used in items 11 to 15 and methodological problems with items 11 (“anxiety about treatment and illness”), 15 (“share … as much as you wanted”) and 17 (“problems addressed”). The adaptation also indicated that patients might have difficulties in interpreting items 8 (“sore or dry mouth”), 10 (“poor mobility”), 11 (“anxiety”), 12 (projected feelings of family and friends), and 14 (“feeling at peace”), thus indicating the need of monitoring during the psychometric validation.

**Conclusions:**

Following this process, IPOS-Fr has proved content and face validity. In our case, the adaptation allowed adjustments to be made to the questionnaire and, when this was not possible, highlighted potential biases and inconsistencies during the validation. The result relied on an intertwined and iterative process of seeking and reaching semantic, conceptual, and normative equivalence. We are now assessing the psychometrical properties of IPOS-Fr.

## Background

In the context of health care, outcome relates to the achievement or failure to achieve desired and appropriate goals. The use of outcome measures can therefore help in determining whether a treatment or intervention is worthwhile and has implications for defining which health care services are the most adequate. In the domain of end of life, specific and capital indicators of palliative care have already been the object of important work and development; for example concerning the topics of meaning in life [1] and quality of life [2]. Significantly less attention has been given to the developing indicators for the assessment of palliative patients’ needs and the quality of care they receive.

To this purpose, the Palliative care Outcome Scale (POS) was developed and validated for use for improving outcome measurement by evaluating several essential outcomes in palliative care: physical and psychological symptoms, existential considerations, family and relatives’ needs, aspects of communication, and practical concerns. The POS has demonstrated construct validity, test/re-test reliability, and internal consistency [3]. The POS has been adapted into many languages such as German [4], Portuguese [5], Indian-Malayalam [6], and Spanish [7, 8]. Since its validation, the POS has been used for clinical and research purposes in cancer centres, nursing homes, and hospice settings [9–13].

The Integrated version of POS (IPOS) unites the main aspects from POS, POS-S (POS Symptom list), and the APCA POS (developed for use in Africa), and has good comprehension and acceptability [14]. IPOS exists in a version for staff and one for patients; it can be completed with a 3 or 7 day recall period. The IPOS is and was adapted in other languages as well, such as German [14], Portuguese [15], Swedish [16], and Japanese [17].

We aimed to cross-culturally adapt and translate IPOS so as to obtain a version with content and face validity in French. For the good development of palliative care research in the French context, it is crucial to have a reference tool recognized internationally for the assessment of the quality of palliative care and patients’ needs. Our specific objectives here are to describe the stages of the cross-cultural adaptation, to highlight the main difficulties encountered, and to review some strategies that would enable them to be circumvented or solved. We equally discuss how the process of trans-cultural adaptation has an impact on the validity of the instrument, and how certain hypothesis can already be formulated in regards to its psychometric properties.

As defined by Epstein and colleagues [18], the purpose of cross-cultural research is to explore the same question among cultures while being aware and respectful of cultural diversity. It therefore requires the distinction between translation (the single process of producing a document from a source version in the target language) and adaptation (the process of considering any differences between the source and target culture). Three dimensions of equivalence are of importance when aiming to adapt a questionnaire to another culture and language [19]: (i) the *semantic equivalence,* concerned with the actual translation*;* (ii) the *conceptual equivalence* concerns the concept that is related to each item used in the questionnaire; (iii) the *normative equivalence* concerns the degree to which the norms of the original and target language may influence the content and structure of the questionnaire. Epstein et al. [18] identified no less than 31 guidelines for the cross-cultural adaptation of self-reported questionnaire, but no evidence of a gold standard. Similarly, a gold standard is also lacking regarding criteria for reporting on the translation and adaptation process of questionnaires on quality of life [20, 21].

## Methods

### IPOS tool

IPOS is composed of 10 questions. Its 17 items are scored with a Likert scale (0–4) and can be considered independently, as well as in subscales (physical symptoms subscale, items 1–10, range 0–40; emotional symptoms subscale, items 11–14, range 0–16, communication and problems subscale, items 15–17, range 0–12), or summed to yield a total score from 0 to 68.

### Translation guideline

**Fig. 1:**
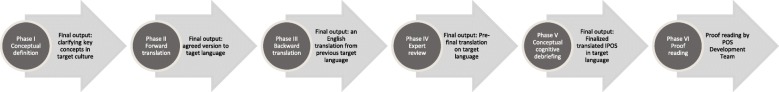
The six phases for reaching trans-cultural adaptation [22]

The increasing interest in POS measures has prompted the POS development team to produce a guidance manual for the cross-cultural adaptation and validation of the POS and all of its derivatives [22]. The POS manual is based on the guidelines provided by the European Organisation for Research and Treatment of Cancer and the Mapi Institute (Fig. 1).

### Phases for reaching trans-cultural adaptation and procedure

#### Phase 1: achieving conceptual definition or equivalence

The purpose of this phase consisted in appraising and clarifying the concepts underlying IPOS items to ensure their equivalence, i.e. their potential to reflect concepts appropriate to the target culture. This phase allowed the research team to become better acquainted with the instrument but also to track down any problems in interpreting and translating certain items. The phase consisted of two steps.

- Step 1. A literature review of existing English to French translations and adaptations of questionnaires regarding palliative care and quality of life, validated or not, identifying which IPOS items have a variable translation in French and may be difficult to translate.
- Step 2. Identification and investigation of key concepts underscoring IPOS items through semi-structured interviews with palliative care staff and patients. The purpose of this step was to assess, prior to the translation, whether and how the target population recognized and understood the IPOS items. Five palliative staff members (two physicians, two nurses, and one psychologist) and five hospitalized palliative patients were interviewed within our service. In order to render the discussion possible, we provided participants with an initial translation based on the results identified from the literature review (Step 1). The interviews lasted between 10 min to 1 h, were audio-recorded and transcribed.

#### Phase 2: a parallel blind forward translation

The purpose of the forward translation is to produce an instrument equivalent between the original and target culture, after a thorough investigation of meanings and interpretations of each item. Also known as the “parallel blind technique”, forward translation consists of two translators who work independently, and whose translations are then checked against each other’s to reach a final version [19]. Written guidelines instruct the translator to consider the items’ semantic, conceptual, and normative equivalence and keep a track of the difficulties and alternative translations when required. In our process, one of the translators had knowledge of palliative care and medical concepts, while the second did not. The two forward translations resulting from this process were compiled by the translation mediators. Dissimilar items were discussed with the translators to reach a common version.

#### Phase 3. A parallel blind backward translation

Backward translation refers to the translation of the instrument from the target language (i.e. as achieved through the forward translation) back to the source one [23]. The purpose of this phase is to do a validity check and ensure that the translation obtained is accurate when compared to the instrument in the original language, and to identify and deal with inconsistencies and conceptual errors.

The back translators worked independently, were bilingual and from different professional backgrounds (one working on healthcare issues, while the other was unfamiliar with this domain). Both were informed that they would be doing a backward translation and were blinded to the original version. The mediators compiled the two obtained translations, compared them to the original, and discussed and resolved with the translators any discrepancies.

#### Phase 4. Expert review

Our French translation was reviewed by a panel of experts formed by members of the palliative research group within our service (nurses, physicians, psychologists), in order to discuss and resolve ambiguities. The result was a pre-final version of French IPOS to be pre-tested with the target population.

#### Phase 5. Cognitive interviewing with palliative care patients and staff

Cognitive interviewing or debriefing corresponds to the qualitative pretesting of an instrument: a purposive sample of the target population completed the questionnaire while using the “think-aloud” technique, discussing and explaining their answers and understanding of the questions and items [24]. Their answers are an indicator of quality in the content validity of the instrument and allow to check for misunderstandings and inconsistent interpretations in the target audience.

Five hospitalized palliative patients and five palliative staff members (one psychologist, two nurses, two physicians) within our service participated to the cognitive debriefing. The interviews lasted 15–60 min, were audio-recorded and transcribed. Modifications were made to the pre-final version of French IPOS as result of these interviews.

#### Phase 6. Final expert review and validation with the creators of IPOS

The translated instrument was again presented for an expert review within our service, and subsequently submitted to the POS development team at Cicely Saunders’ Institute together with a report on the stages of translation. After ensuing discussions, further modifications were made to the translation. The final translation was used for the psychometric validation.

The six phases described above were realized between May 2016 and February 2017.

## Results: findings and challenges from the French translation

### Challenges highlighted in phase 1 (conceptual definitions)

The purpose of this phase was to identify incoherencies throughout existing translations from English to French of IPOS terms contained in questionnaires on quality of life and palliative care. In Step 1 of Phase 1, we explored how IPOS items appearing in ten other English-language questionnaires were translated into French (see Table 1).

**Table 1:** Alternative translations for three IPOS items

IPOS Item	French translations in similar questionnaires
Anxiety	*Anxiété* (QUAL-E^a^, ESAS^b^)
	*Angoisse* (STAS^c^)
Being depressed	*Déprime/Sentir déprimé* (STAS, ESAS, EORTC^d^)
	*La dépression* (QUAL-E)
	*Sentir triste* (MQOL-R^e^)
Shortness of breath	*Essouflement* (QUAL-E, MDASI^f^)
	*Souffle court* (EORTC)
	*Peine à respirer* (ESAS)

^a^Quality of Life at the End of Life: QUAL-E [25]

^b^Edmonton Symptom Assessment: ESAS [26]

^c^Support Team Assessment Schedule: STAS [27]

^d^EORTC Quality of Life of Palliative Cancer Patients: EORTC QLQ-C15-PAL [28] ^e^McGill Quality of Life Questionnaire-Revised: MQOL-R [29]

^f^M. D. Anderson Symptom Inventory: MDASI [30]

The results showed that most items are translated the same way throughout all questionnaires. However, for the items related to “anxiety”, “depression”, and “shortness of breath”, several translations exist, sometimes bearing discordant meanings.

Being aware of these alternative translations allowed us to explore further, in Step 2 of the Phase 1, the various meanings of IPOS items during interviews with palliative staff and patients; those fluent in English were asked how they would translate a certain item. For example, regarding the translation of “shortness of breath”, two staff members differentiated between *peine à respirer* (the adequate translation according to them, defined as the sensation of discomfort when breathing) and *souffle court*, which is more activity-related and comprised within *peine à respirer*. Regarding the translation of “anxiety”, four staff members acknowledged the recurrent use of both *anxiété* and *angoisse* when talking to patients but identified *anxiété* as being the most adequate, as *angoisse* is more diffuse and profound. Regarding the translation of “feeling depressed”, four staff members recommended the use of *se sentir déprimé*, distinguishing it from *dépression*, a more clinical term.

### Challenges highlighted in phase 2 (forward translations)

Both forward translations were in agreement most of the time. Overlooking grammar and syntax variation, dissimilar translations were found in relation to formulations contained in IPOS’s seventeen items (item 2 “shortness of breath”, item 3 “lack of energy”, item 6 “poor appetite”, item 8 “sore mouth”, item 10 “poor mobility”; items 11 and 12 “feeling anxious”; item 14 “felt at peace”; item 15 “share how you are feeling”; item 17 “problems ( …) addressed”) and two Likert response options; two of these (“shortness of breath” and “feeling anxious”) had already been highlighted during the literature review as being the source of alternative translations. The fact that one of the translators was a palliative care physician proved very useful, as she could sometimes enlighten on the word most used with her patients. Taking into account the literature review and the preceding interviews, joint discussion between the translators and the translation mediators allowed to reach consensus for all except two items (“poor mobility” and “problems addressed”). We show below changes made that reveal how this phase does not consist in a mere semantic translation.

#### Item 5 – “vomiting (being sick)”

In the English version of IPOS, two items of the physical symptom subscale have explanatory parenthesis: “nausea (feeling like you are going to be sick)” and “vomiting (being sick)”. Both translators found that the explanatory brackets could be waived for “vomiting”, as it contains an idiomatic expression which doesn’t have an equivalent in French, while the term “vomiting” (*vomir*) is frequently used in French and less so in vernacular English; preliminary interviews with patients and professionals (Phase 1, Step 2) also confirmed this. With the POS’s team agreement during the Expert Review (Phase 4), the explanatory bracket for item 6 was waived in the staff and patient IPOS French translation.

#### Gender-inclusive language

A second challenge concerned using a gender-neutral vocabulary in French (in the English version, staff IPOS uses both masculine and feminine gender - “s/he”-, while patient IPOS is neutral). The issue became obvious during the forward translation, as one of the translators used only the masculine for patient IPOS, while the other used both. The hospital’s communication service advised to use the masculine with an explanatory footnote (“In order to facilitate the reading of the document, the generic masculine form is used to designate both genders”).

#### Syntactic inconsistencies

During the forward translation, it became obvious that IPOS, in its original version, did not maintain syntactic coherence on items 11 to 15. In the staff version of IPOS items 13 and 14 are formulated as a possibly uncertain evaluation (“Do you think s/he felt …”) while items 11, 12, 15 are more categorical (“Has s/he/the patient been …”?). Furthermore, both patient and staff IPOS use two different wordings to refer to feelings of anxiety: in item 11 it’s a matter of “feeling anxious” while in item 12 it’s of “being anxious”. While minor, such discrepancies, when not supported by an interpretation guide, can be misleading and introduce a bias in the evaluation (this was confirmed by cognitive interviews with staff). Upon consultation with the POS development team, it resulted that this incoherence was due to how IPOS was compiled from various POS versions. We maintained the same grammatical formulations in French, granting they would be changed once a review of original IPOS would be made.

The forward translation (Phase 2) resulted in semantic modifications for items 5, and 11 to 15, which gave way to a first version of French IPOS faithful to the meaning of the items contained in the original version but also adapted to the specificities of the target language.

### Challenges highlighted in phase 3 (backward translations)

Reflecting the choices made previously, both backward translations were very close to the original instrument. In four cases (one item and three Likert response options) only one of the backward translations matched the original IPOS. Neither of the backward translations matched for four items (item 2 “shortness of breath”, item 8 “dry mouth”, item 10 “poor mobility” and item 17 “problems (…) addressed”) and four Likert response options (“severely”, “overwhelmingly”, “occasionally”, “hardly”). Three of these (“shortness of breath”, “poor mobility”, and “problems addressed”) were already highlighted from the forward translation as potentially problematic. We discussed these dissimilarities with the translators in order to find the best alternative.

### Challenges highlighted in phase 4 (expert review)

Our expert review served two purposes: to clarify two translations which remained unresolved (items 10 “poor mobility” and 17 “problems addressed”) and to highlight further methodological problems and potential biases related to the language used in the original questionnaire (item 11 and 15, and a Likert response options for item 17).

#### Item 10 – “poor mobility”

The translation problem was due to the negative formulation of the item but also to the numerous terms available to refer to a problematic mobility in French. Furthermore, the literature research (Phase 1) could not be of any help as no other questionnaires contained this item. The item was initially translated during Phase 2 by *faible mobilité* and *réduction de votre mobilité/mobilité réduite*. Both translations were tested with patients and staff and no important differences were noted in their comprehension. Two staff proficient in English and French also suggested alternatives (*mobilité restreinte, mobilité limitée, mobilité diminuée*). After discussion with our translators, we selected the term *mobilité réduite* that, according to them, designates a handicap that leads to reduced mobility and limited or no autonomy in movement. During Phase 3, both back-translators translated the item as “reduced mobility”, which did not match the original formulation. Nevertheless, when asked, back-translators also opted for *mobilité réduite*.

#### Item 11 – “anxiety about illness or treatment”

The expert review highlighted that item 11 (“Have you been feeling anxious or worried about your illness or treatment?”) targets two dimensions that would each require their own evaluation: anxiety regarding illness vs anxiety regarding treatment. This was equally noted by one patient (“Maybe the difference is between illness and treatment. Illness is one thing but the treatment…”) and one staff (“We talk of two different things under the same heading”). The POS development team also agreed that such formulation may generate a bias. Nevertheless, given that it was not possible to remove one item or insert an additional question, we decided to keep the wording of the item, while keeping track of this potential bias during the cognitive interviews and psychometric validation.

#### Item 15 – “as much as you wanted”

It was equally felt by some expert review members that item 15 (“Have you been able to share how you are feeling with your family or friends as much as you wanted?”) might introduce a bias, as it is fashioned from the standing point that the patient had wanted to share feelings (which may not necessarily be true). Nonetheless, as for item 11, the only possibility to rectify the bias was to alter the question or split it in two. We decided to monitor potential bias during the cognitive interviews and psychometric validation.

#### Item 17 – “problems addressed”

The translations were also inconclusive concerning item 17, “problems addressed”, the issue at stake being the meaning of the term “addressed”, which was forward-translated into French as *aborder tout problème* (to relay any problem) or *trouver une solution aux problèmes* (to find a solution to the problems). In order to maintain the duality of the formulation, we opted for the translation *pris en compte* (taken under consideration), which, while making clear that the problem had been addressed, did not specify whether it had been solved. This was back-translated by “considered” and “taken into account”, terms that, according to the expert review, were dissimilar to the original term. The POS development team clarified the meaning of the item and advised to opt for the translation *problèmes résolus* (problems resolved).

#### Likert response options– “problems addressed/no problems”

Another issue that was brought up and resolved during the expert review concerned item 17 (“Have any practical problems resulting from your illness been addressed?”), for which one of the Likert response options is “problems addressed/no problems”. Members of the expert review noted that the compounded response might be misleading in the clinical setting, since it is relevant for a clinician to differentiate between the two options. Nevertheless, it was felt that, in order to establish a score that reflects actual palliative needs of patients, a situation in which there are no problems is equivalent to one in which problems have been addressed – in both cases, there is no need for care; for validation purposes, both answers could therefore be evaluated by the same score.

### Challenges highlighted in phase 5 (cognitive interviews)

The aim of this phase was identifying comprehension difficulties through cognitive interviews. In general, patients and staff agreed on item significance and their definitions were similar, though patients were aware that a symptom’s interpretation is subjective. The heightened subjectivity of patient evaluations might explain a potential low agreement when comparing patient and staff IPOS versions during the psychometric phase.

#### Symptom evaluation

Some patients had difficulties and even refrained from evaluating symptoms unrelated to their illness. For example, one patient notes that constipation was a life-long problem yet not a consequence from their illness (*“constipation is a problem in the way I’m built that I’ve always had, so this is a daily struggle (…) I can’t say it’s the illness, it’s something I had in me before being ill”*) while another explains that his increasing drowsiness is not induced by the illness but rather by the context (*“Of course I’m drowsy. If you had visits and you had 5-6 people at the same time, you’d end up drowsy, you can’t answer to everybody”*). Both patients scored these symptoms low. Based on these cases, it is possible that some patients might give low scores to symptoms that affect them but are not perceived as a consequence of their illness.

Furthermore, one patient reflected on the difficulty of making evaluations on a 3-day scale rather than considering the whole duration for which the item was problematic. For symptoms lasting up to a week it is possible to use the 7-day IPOS; nevertheless, it seems important to remind that IPOS is a tool meant to ensure responsiveness to current and arising problems.

One staff commented on the lack of an option “I don’t know”, instead of “impossible to answer”, given that the meaning is not necessarily identical. Again, in order to maintain coherence with the original language instrument we did not alter the formulation, but we noted that this formulation might account for staff lack of response.

#### Item 8 “Sore or dry mouth”

While dryness of mouth was well understood by patients, some of them had difficulties in understanding the first part of the item - “sore mouth” (*bouche douloureuse*). In our case, one patient associated it to having a toothache and another to having “small sores”, but most just said that it did not concern them. In order to highlight the part of the item to which most patients relate, we decided that in French it is more judicious to switch the adjectives (“dry or sore mouth”).

#### Item 12 - projected feelings of family or friends

Patients expressed difficulties with item 12 which requires them to project what other people’s feelings are: “*I’m not in their head nor in their hearts*”; “*I have no idea (…) I don’t know what they think*”. This might account for missing data during the psychometric validation.

#### Item 14 - “Feeling at peace”

Three staff members identified a difficulty regarding item 14, “Do you think s/he has felt at peace”, related to their habit of asking it (“*This one here is not a question that I have the habit to ask*”; “*It’s something I wouldn’t ask*”) and its adequate timing (“*We can ask this question but it means we’ve already explored the question of spirituality, of what he expects from death*”). They reflected on how it could be formulated, noting that it might be conceptually incomplete (“*there’s a bit of the phrase that is missing in French. Do you feel at peace… with yourself, with others or with this situation, with death or with your illness*”), and offered alternatives such as “tranquility” (*être tranquille*) and “being serene” (*être serein*). The alternatives were tested during cognitive interviews with patients, who best recognized and referred to *être en paix* (“being at peace”). This term was coined during forward and backward translations.

#### IPOS use by mobile teams

A dimension explored by staff concerned IPOS applicability to/by palliative mobile teams. Staff noted that, contrary to hospital units, mobile teams do not monitor the patients daily, which makes it difficult to evaluate the evolution and importance of non-physical items (11 to 17). Staff equally pointed out that mobile team staff do not necessarily discuss the patients’ contact with kin (items 12 and 15) and that the non-physical aspects (items 11–17) can only be discussed after establishing a trusting relationship.

### Overall impression of participants

Patients found that the questionnaire was easy to respond to and the questions were easily understandable and appropriate; only one patient noted that some questions require time to think before answering. One patient suggested inserting a thank you note (a “diplomatic formula”) at the end of the questionnaire, which was implemented. Staff found the questionnaire interesting and useful; only one staff was slightly critical regarding the use of questionnaires as means for assessing care outcomes and patients’ needs.

## Discussion

The first part of our study consisted in translating and culturally adapting IPOS patient and staff versions to French. The iterative process of the trans-cultural adaptation allowed the creation of a translated version of IPOS with demonstrated face and content validity for both patients and staff. Our work was guided by a manual issued specifically for the adaptation of POS, itself based on standardized methodology [22, 31].

The complexity of the cross-cultural adaptation may increase with the difference between the two languages and cultures. The adaptation process revealed that the most important linguistic cultural differences between the Swiss francophone population and the English one consist in wording of the questions, and particularly in the use of metaphors and expressions to identify concepts; while for item 5 (“vomiting”) this was easily addressed, it required consultations with our translators and expert review for item 10 (“poor mobility”).

To date, two other IPOS translation process have been published, in Portuguese [15] and Swedish [16]. We were comforted by the finding that the Swedish team encountered similar challenges to ours, such as the translation of “being depressed” (which was replaced with the Swedish term for “gloomy”), “shortness of breath”, “being at peace” (replaced with the Swedish term for “calmness and stillness”), and “problems addressed” (which was modified to clarify its meaning). The Swedish version also identified problems in the formulation of item 15 (“Have you been able to share how you are feeling with your family or friends as much as you wanted?”) which may generate misunderstandings, though the phrasing was left the same. The Portuguese version identified issues with verb tenses, though the changes regarded especially formatting; the explanatory brackets for item 5 (“vomiting”) were also waived.

The review of translated instruments (Phase 1) displayed the wide semantic variation for some of IPOS’s items. Such variations may prejudice the quality of international comparisons, correlations with other questionnaires, and ultimately the questionnaire’s validity. This frequent lack of coherence is noted by other researchers investigating questionnaire adaptation as well [32], and can here be explained by the linguistic territory (French language variation between France, Canada, Belgium, or Switzerland, but also within a country) but also by the fact that several of these translations have not been culturally adapted and/or validated in a rigorous way. This stresses the importance of validating questionnaire translations, the need of taking into account previous translations, and of providing a written journal documenting the choices made.

The methodology and analysis of exploratory and cognitive interviews with patients and staff is another important resource for cross-cultural adaptation. Even after a rigorous translation, it was important to have the patients’ and staff’s impression and experience of IPOS items. In our case, patient and staff interviews allowed investigation of several translation alternatives, choosing the most appropriate, as well as understanding the extent of potential biases (such as the formulation of item 16).

Transcultural adaptation is an important stage in the translation of a questionnaire and may directly affect the validation step. In our case, the adaptation allowed adjustments to be made to the questionnaire and, when this was not possible, highlighted potential biases and inconsistencies during the validation. The result relied on an intertwined and iterative process of seeking and reaching semantic, conceptual, and normative equivalence. An example of a challenging adaptation on the semantic level concerns the item “poor mobility”, for which we had to test and discuss several formulations even though the concept to which it referred to was very clear. On the conceptual level, “problems addressed” required an investigation of the meaning associated to this item. The normative dimension was underlined by the patients themselves, for example when expressing difficulty in evaluating how other people feel (with regard to item 12, “have any of your family or friends been anxious or worried about you?”), which can account for low response rate and for low inter-rater agreement during the psychometric validation of the instrument. Regarding IPOS’s clinical applicability, cognitive interviews revealed that while staff finds the questionnaire useful and interesting, some find that non-physiological items cannot be evaluated on the first encounter with the patient.

An interesting reflection that can be made in the aftermath of our adaptation process concerns the range of changes that not only *should*, but *can*, be made to the questionnaire. With cross-cultural translation requiring an attention to fine linguistic details over an important span of time, the process equally brings to light potential problems with the instrument in the original language – whether on the semantic, conceptual, or normative level. In a way, it may be that more attention is given to the creation of a questionnaire by means of a validated adaptation from another language, than to its creation from scratch. Our experience of IPOS’s translation showed that while being able to identify potential weak points in the original instrument, such as for item 12 (anxiety “about your illness or treatment”), which requires an assessment of two potentially different aspects and may generate confusion, we were not able to change items in order to remain faithful to the original conception of the questionnaire.

Two factors may appease a researcher’s conscience. First, identifying such biases is important for explaining shortcomings in the psychometric validation of the translated instrument, which are not due to a lack of quality in the translation but to the questionnaire itself. Second, keeping track of such biases and informing the team having created the original instrument can become part of an ongoing effort to improve said instrument and produce revised versions. For example, the MQOL-R (the revised version of the MQOL) improved the instruments’ psychometric properties and feasibility [29].

### Limitations

Several limitations can be noted regarding our methodology. First, due to time limitations, we restricted our literature review (Phase 1 Step 1) to English-to-French translations of questionnaires frequently used in palliative care; nevertheless, an exhaustive literature review regarding all existing translated questionnaires might have contained more clues as to the most used translations for IPOS items such as “mobility” that were particularly hard to translate. Second, we were not able to use translators representatives of our specific target population, as recommended in other guides [31]. Third, we included in our interviews with staff only three professions (physicians, nurses, and psychologists). The inclusion of other professionals specialized in palliative care, such as assistive personnel, social workers, or chaplains, might have provided even more information regarding the meaning and adequate translation of IPOS items whose conceptualization is ambiguous, such as “being at peace”. Last but not least, due to the wide spread of French language, the choices we made in our adaptation might not be pertinent for other linguistic areas such as Belgium or Francophone Canada.

## Conclusion

The present article highlights the main challenges faced during the trans-cultural adaptation of IPOS to French. The main result of this adaptation process was the production of a French version of staff and patient IPOS with proven acceptability and face validity among our target population, which is currently being validated.

The adaptation resulted in changes made to the formulation of items and in the identification of biases that can impact the psychometric validity of the questionnaire, in regards to items 11, 15, and 17. Given the need for a translation to remain as close as possible to the original instrument, some of these biases could not be addressed. Their identification was nevertheless important, as it can explain problems in psychometric validity with specific items; these issues were reported to the POS development team and can be taken into consideration when a revised version of IPOS will be made.

Beyond its relevancy for the translation of IPOS, our comments address the basic process of producing a cross-cultural translation. In particular, we stress the importance of following specific or general guidelines regarding questionnaire translation, whose absence may generate lower reliability and lower correlations than in the original version [33].

Another important factor influencing the quality of an adaptation is keeping an updated journal of the process whenever a translation is made of an instrument, and the relevance of producing an interpretation guide for the items used in any questionnaire, whether for the original language or for a translated version. Equally important to our mission was the continuous dialogue maintained with the POS development team who produced the POS and the IPOS. Our exchanges with the POS development team allowed to clarify the import of the guidelines provided in their cross-cultural adaptation manual and provide solutions when needed. For example, they provided support in defining how the interviews should best take place (whether individual or as focus groups), how to orient our literature review, and aided us regarding the extent of modifications we can make in comparison to the original version of IPOS. Nevertheless, we equally argue that a document explaining the choice of words and their meaning for the original instrument could have been very useful as well. Behling and Law [19] also note that questionnaires can be, from the start, created in several languages or at least “written with translation in mind” (for example, avoiding metaphors and regional colloquialisms).

## Abbreviations

APCA POS: African Palliative Care Association Palliative care Outcome Scale
EORTC QLQ-C15-PAL: Quality of life of palliative cancer patients
ESAS: Edmonton Symptom Assessment
IPOS: Integrated Palliative care Outcome Scale
MDASI: M. D. Anderson Symptom Inventory
MQOL-R: McGill Quality of Life Questionnaire-Revised
MyPOS: Myeloma-specific Palliative care Outcome Scale
POS: Palliative care Outcome Scale
POS-S: Palliative care Outcome Scale Symptom list
QUAL-E: Quality of Life at the End of Life
STAS: Support Team Assessment Schedule
